# DNA-triggered activation of aptamer-neutralized enzyme for *in situ* formation of injectable hydrogel†

**DOI:** 10.1039/d5nh00314h

**Published:** 2025-06-16

**Authors:** Connie Wen, Yixun Wang, Kyungsene Lee, Xuelin Wang, Yong Wang

**Affiliations:** a Department of Biomedical Engineering, The Pennsylvania State University, University Park PA 16802 USA yxw30@psu.edu

## Abstract

Injectable hydrogels have been widely studied for the embolization of vascular malformations and the control of bleeding in hemorrhages. An ideal injectable hydrogel in these applications needs to form once contacting with the blood components, which enables easy control of hydrogel formation and injectability. However, this type of injectable hydrogel has not yet been widely studied. In this work, an injectable hydrogel system was developed by using a bispecific aptamer-neutralized enzyme and a triggering DNA. The results show that the system remained in its solution or pre-gelation state in the presence of the bispecific aptamer. Upon contact with the triggering DNA, the system was transformed into a hydrogel state. *In vitro* aneurysm and endovascular embolization were further conducted, and the results showed the DNA administered out of the hydrogel system could trigger the activation of aptamer-bound enzymes for the accelerated formation of the injectable hydrogel. Therefore, this study has successfully demonstrated that a bispecific aptamer-neutralized enzyme in the pre-gelation system can be rapidly released to accelerate the formation of injectable hydrogels when the system is in contact with the blood that contains a triggering DNA.

## Abstract 2

An injectable hydrogel system was developed by using a bispecific aptamer-neutralized enzyme and a triggering DNA.

## Graphical abstract

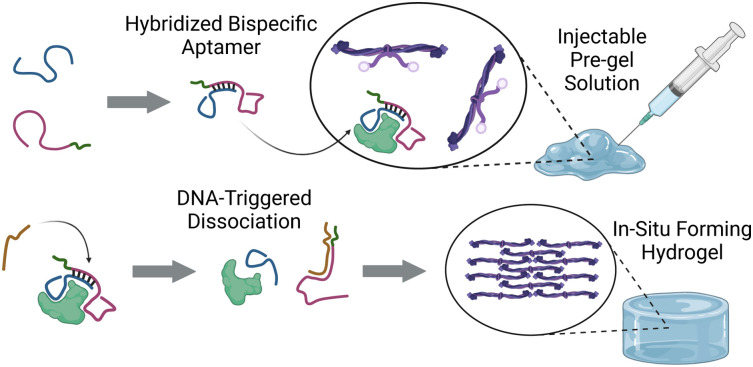

**New concepts**

An ideal blood-contact injectable hydrogel should be easily injectable but rapidly form a stable hydrogel upon contact with blood. However, designing such a hydrogel remains challenging. We utilized bispecific aptamers and their complementary sequences to regulate the injectability and gelation kinetics of hydrogels. The bispecific aptamers were designed in a hybridized form to exhibit strong enzyme neutralization, thereby inhibiting gelation and enabling easy injectability. Upon reversal of this inhibition by DNA triggers, rapid hydrogel formation was initiated. This study demonstrates the feasibility of using bispecific aptamers and DNA triggers to engineer injectable and controllable *in situ* hydrogels for potential applications such as vascular embolization therapy and hemostasis.

## Introduction

Injectable hydrogels, owing to their minimally invasive delivery, have been widely studied for biomedical applications ranging from the delivery of cells or drugs,^1–5^ to serving as bioadhesives.^6–8^ Injectable hydrogels have also been investigated for applications involving direct blood contact, including the embolization of vascular malformations and their use as hemostatic biomaterials to control bleeding.^9–13^ Their ability to conform to irregular shapes allows them to be delivered directly to vascular lesions or locations with vascular damage, effectively sealing or occluding the area of disease. Ideally, injectable hydrogels exist as solutions or exhibit shear-thinning properties, becoming less viscous under shear to promote high flowability. This is a critical characteristic for successful injection *in situ*. The *in situ* gelation can be achieved by using different stimuli such as temperature,^14,15^ pH,^16^ light,^17,18^ and many other factors.^19^ However, most *in situ* injectable hydrogels form abruptly, which causes the difficulty of injection and penetration into microvasculature.

Fibrin hydrogels have been studied as sealants for wound closure and bleeding control.^20–22^ Due to the rapid reaction between fibrinogen and thrombin, fibrin hydrogels are typically delivered using a dual-barrel syringe system, where fibrinogen and thrombin are stored separately and mixed only at the time of administration.^23,24^ Once fibrinogen and thrombin are mixed, gelation is immediately initiated, leading to a rapid increase in viscosity. As a result, the mixture becomes increasingly difficult to inject through a catheter or administered into a deep region. Ideally, a blood-borne trigger can be designed to stimulate the fibrinogen solution and control its gelation kinetics to control the gelation rate of fibrin. Thrombin has two important binding sites, exosite I and exosite II, which can affect substrate recognition and the enzymatic activity of thrombin.^25,26^ The binding of these two exosites with aptamers can regulate the bioactivity of thrombin. In addition, the aptamer–thrombin interaction can be reversed using complementary DNA molecules.^27,28^ Therefore, the complementary DNA may be used as a blood-borne exogenous trigger to regulate the catalytic activity of aptamer-bound thrombin for controlling the formation of *in situ* injectable fibrin hydrogels.

**Fig. 1:**
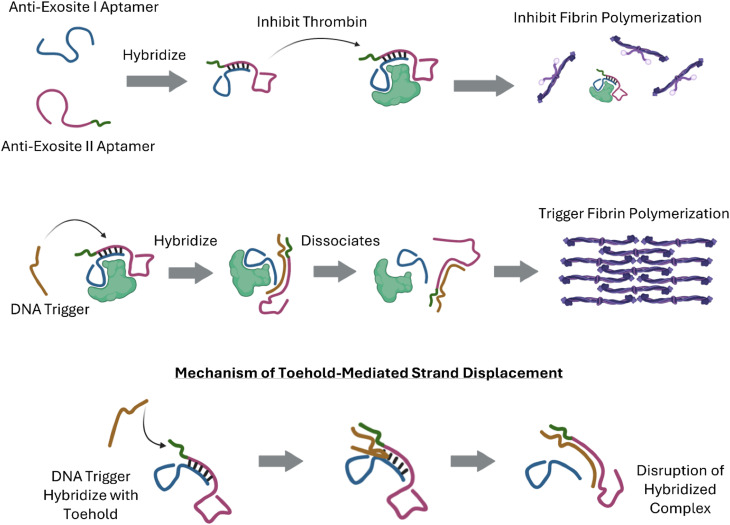
Scheme of bispecific aptamer-mediated thrombin inhibition (top), DNA triggered fibrin formation (middle), and mechanism of toehold-mediated strand displacement (bottom).

In this study, a bispecific aptamer was designed with two anti-thrombin aptamers hybridized together (Fig. 1). The two aptamers including 15-mer and 29-mer bind exosite I and II of thrombin, respectively.^29,30^ The rationale of designing this bispecific aptamer was to strengthen the binding of aptamers and thrombin *via* bivalency instead of monovalency. Moreover, as the two aptamers were linked *via* hybridization rather than covalent bonds, it would be easier for the triggering complementary DNA to dissociate them from thrombin. Experiments were designed to evaluate the binding strength of the bispecific aptamer and thrombin, bispecific aptamer-mediated thrombin neutralization, gelation kinetics and rheology of the reaction solution in the presence and absence of triggering DNA, injectability of the hydrogel system, and *in situ* formation of fibrin hydrogel in two *in vitro* models.

## Results and discussion

### Design of bispecific aptamer and DNA trigger

A bispecific aptamer formed by hybridization of two single-stranded aptamers was designed. The sequences of the two hybridized aptamers in the bispecific aptamer are shown in Table 1. The bispecific aptamer was first constructed with 15-mer and 29-mer, each with 5T spacer at the 3′ end, followed by a 12-nucleotide “sticky region” for hybridization. The binding affinity of the hybrid bispecific aptamer to thrombin was analyzed with flow cytometry in microparticle assay (Fig. S1A, ESI†). The microparticle assay showed that the *K*_d_ was 4.8 nM (Fig. S1B, ESI†).

**Table 1:** DNA sequences of aptamers and DNA Triggers. Underlined: hybridization sequences. Bold: toehold sequence. TH, toehold. DNA trigger to 29-mer-Sticky-TH is complementary to the last 5 bases of 29-mer, 5T spacer, sticky region, and toehold sequence

DNA	Sequence (5′ → 3′)
15-mer-Sticky (exosite I-targeting)	GGTTGGTGTGGTTGG TTTTT G̲A̲C̲ C̲C̲T̲G̲ C̲G̲T̲ A̲C̲C̲
29-mer-Sticky (exosite II-targeting)	AGTCCGTGGTAGGGCAGGTTGGGGTGACT TTTTT G̲G̲T̲A̲C̲G̲C̲A̲G̲G̲T̲C̲
DNA trigger of 15-mer	CCA ACC ACA CCA ACC
DNA trigger of 29-mer	AGT CAC CCC AAC CTG CCC TAC CAC GGA CT
29-mer-Sticky-TH	AGTCCGTGGTAGGGCAGGTTGGGGTGACT TTTTT G̲G̲T̲A̲C̲G̲C̲A̲G̲G̲T̲C̲ **CGTTA**
DNA trigger of 29-mer-Sticky-TH	TAA CGG ACC TGC GTA CCA AAA AAG TCA
ss bispecific aptamer	GGTTGGTGTGGTTGG TTTTTTTTTTTTTTTTTTTTTT AGTCCGTGGTAGGGCAGGTTGGGGTGACT

To reverse thrombin inhibition and initiate fibrin gelation, the complementary DNA sequences to 15-mer and 29-mer (Table 1) were used as DNA triggers to release the hybrid bispecific aptamer from thrombin by disrupting the secondary and tertiary structure of the 15-mer and 29-mer. The microparticle assay showed that the addition of the DNA triggers from molar ratio of 0.5 : 1 to 10 : 1 to hybrid bispecific aptamer only induced about 30 to 45% of aptamer dissociation from thrombin (Fig. S1C, ESI†). These results showed that the DNA triggers directly targeting the 15-mer and 29-mer sequences were inefficient in reversing the inhibition. This could be due to the stable conformation of the hybrid bispecific aptamer,^31,32^ which “locks” the 15-mer and 29-mer into their respective exosites on thrombin, creating a higher barrier for the complementary DNA triggers to access their targets and induce dissociation of the aptamer from thrombin.

To reverse aptamer-mediated thrombin neutralization more effectively, a new DNA trigger was designed to hybridize with the entire sticky region and the 5T spacer on 29-mer strand, plus the last 5 bases of 29-mer from the 3′ end (Table 1). With this design, we expected the DNA trigger to disrupt both the formation of hybrid bispecific aptamer and the tertiary structure of the aptamer. The aptamer structure disruption was chosen for 29-mer instead of 15-mer because 15-mer itself has a low binding affinity.^27,28,33^ To allow for easier hybridization between the sticky region and DNA trigger, a 5-nucleotide long toehold (TH) sequence was attached to the 3′ end of 29-mer-5T-sticky (Fig. S2, ESI†). The toehold sequence “CGTTA” (5′ to 3′) was designed with the following rationale: (1) to have adequate binding affinity and stability;^34^ (2) to avoid interference with the formation of hybrid bispecific aptamer; (3) promote an energetically favorable displacement reaction.^35^ The DNA trigger was then designed to hybridize with the last 5 bases of 29-mer, 5T spacer, sticky region, and the toehold sequence.

The formation of hybrid bispecific aptamer with toehold sequence was confirmed by gel electrophoresis (Fig. S3, ESI†) using FAM-labeled exosite I-targeting strands and unlabeled exosite II-targeting strands. The exosite I-targeting strand was labeled with the fluorophore because the binding of this strand affects the subsequent catalytic activity of thrombin. The hybrid bispecific aptamer without toehold sequence had ∼86% of hybridization efficiency whereas hybrid bispecific aptamer with toehold sequence had hybridization efficiency of ∼78%. The result shows that the existence of toehold sequence had a minimal effect on aptamer hybridization.

### Binding affinity of toehold-incorporated hybrid bispecific aptamer (THBA) association and DNA trigger-mediated dissociation

**Fig. 2:**
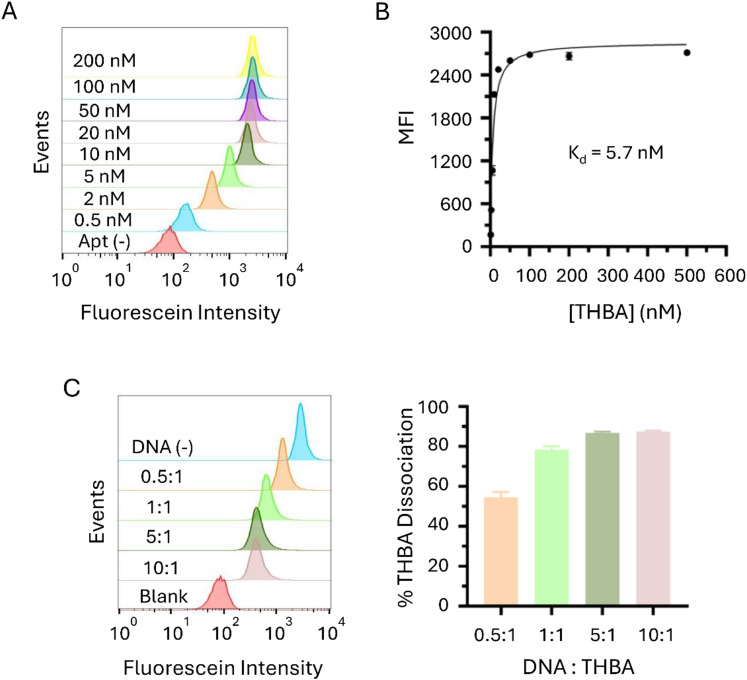
Molecular interaction between toehold-incorporated hybrid bispecific aptamer (THBA) and thrombin with DNA trigger. (A) Flow cytometry of thrombin-bound microparticles treated with varied THBA concentrations from 0.5 to 200 nM. (B) Fitted curve of THBA–thrombin binding. MFI, mean fluorescence intensity. (C) Flow cytometry analysis of DNA-triggered THBA dissociation from thrombin on the microparticle surface. The concentration of THBA was 500 nM, and the molar ratio of DNA trigger to aptamer was varied from 0.5 : 1 to 10 : 1.

The binding affinity of THBA was determined using a microparticle assay. The FAM-labeled 15-mer-5T-Sticky was hybridized with 29-mer-5T-Sticky-Toehold to generate a FAM-labeled hybrid bispecific aptamer with toehold sequence (FAM-THBA). Different concentrations of FAM-THBA, ranging from 0.5 to 200 nM, were incubated with thrombin-immobilized microparticles, followed by flow cytometry analysis to determine the mean fluorescence intensity (MFI) of the microparticles (Fig. 2(A)). The MFI was plotted against different aptamer concentrations and the curve was fitted. The dissociation constant of THBA was estimated to be around 5.7 nM, which is slightly higher than the value (*K*_d_ = 4.8 nM) of hybrid bispecific aptamer without toehold sequence (Fig. 2(B) and Fig. S1B, ESI†). This shows that the incorporation of the toehold sequence on 29-mer strand did not significantly decrease the binding affinity of the hybrid bispecific aptamer. When compared to the dissociation constant of the single-stranded bispecific aptamer containing 10T spacer,^27^ the dissociation constant of THBA was higher. This might be due to the non-covalent nature of the linker formation.

To assess the dissociation of THBA mediated by the DNA trigger, DNA triggers of different molar ratio to THBA (0.5 : 1, 1 : 1, 5 : 1 and 10 : 1) were added to THBA-bound thrombin conjugated microparticles. The DNA triggers designed to hybridize with the sticky region on 29-mer strand showed higher efficiency in causing THBA dissociation from thrombin. DNA trigger at 0.5 : 1 molar ratio to THBA resulted in about 50% of THBA dissociation (Fig. 2(C)). With the molar ratio of 5 : 1, 80% of THBA were dissociated from thrombin. Since the dissociation was quantified based on the fluorescein intensity, and fluorescein was labeled on the exosite I-targeting strand, a decrease in fluorescein intensity is a direct result of dissociation of the exosite I-targeting strand. These results show that the disruption of the hybridization between the 15-mer strand (exosite I-targeting) and 29-mer strand (exosite II-targeting) effectively caused the dissociation of 15-mer from thrombin. This was most likely due to the decoupling of 29-mer from 15-mer, which left the relatively weak bounded 15-mer strand to be easily dissociated from thrombin.

### THBA-inhibited and trigger-induced fibrin gelation

**Fig. 3:**
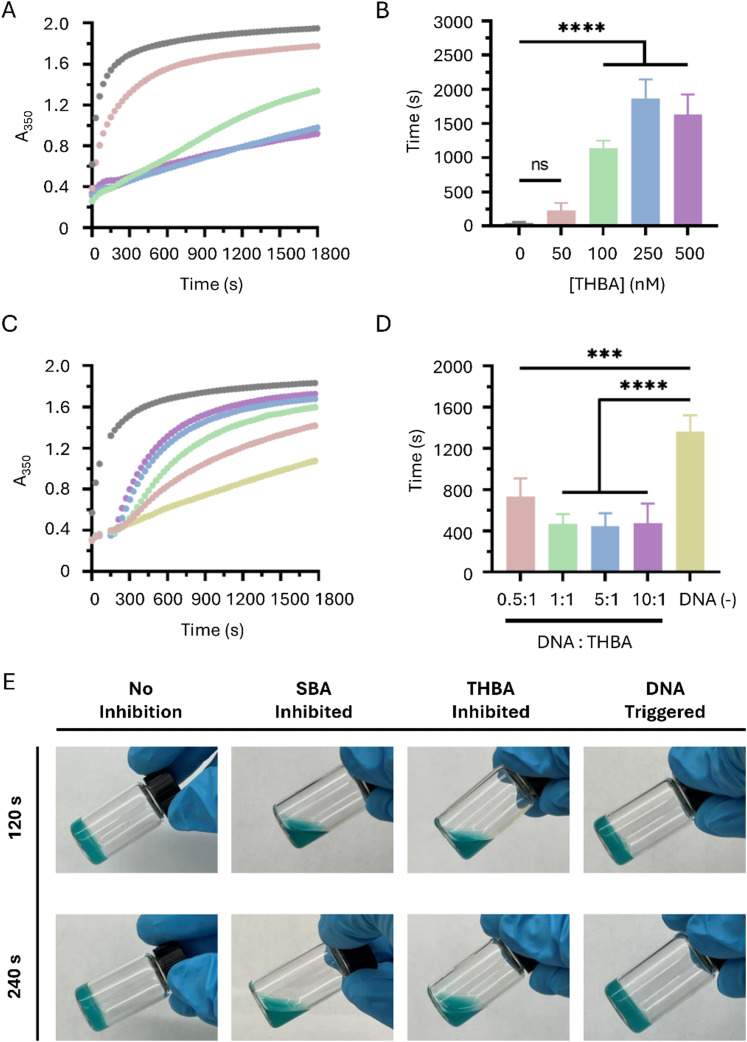
Effects of THBA and DNA trigger on kinetics of fibrin formation. (A) Turbidity of fibrinogen and thrombin solution in the presence of THBA at different concentrations (from the top to bottom: 0, 50, 100, 250, and 500 nM). (B) The time of the solutions reaching 50% of the maximum turbidity, *n* = 3. (C) Turbidity kinetics of fibrin formation in the presence of aptamer (250 nM) and DNA trigger. The DNA trigger-to-aptamer ratios from the top to the bottom were no aptamer, 10 : 1, 5 : 1, 1 : 1, 0.5 : 1, and no DNA trigger, respectively. (D) The time of the solutions reaching 50% of the maximum turbidity after the addition of DNA trigger, *n* = 3. (E) Formation of fibrin hydrogels in a vial after different treatments. The aptamer concentration was 250 nM. The DNA trigger-to-THBA molar ratio was 10 : 1. The fibrinogen concentration was 25 mg mL^−1^, and the thrombin concentration was 2.5 U mL^−1^. SBA, single stranded bispecific aptamer (*p ≤ 0.05, ***p ≤ 0.001, and ****p ≤ 0.0001).

A turbidity assay was used to examine fibrin gelation catalyzed by the THBA-inhibited thrombin. At 50 nM of THBA, a mild inhibition in fibrin gelation was observed. When the concentration of THBA increased to 100 nM, the gelation was significantly inhibited. Moreover, a further increase in THBA concentration to 500 nM prolonged the inhibition in fibrin gelation (Fig. 3(A)). The time when the pre-gel solution reached 50% of the maximum absorbance was recorded and compared between different concentrations of THBA. When the concentration of THBA was 50 nM, the time required to reach 50% maximum absorbance was not significantly different from non-inhibited control. However, a significant difference in time was observed when THBA concentration increased to 100 nM (Fig. 3(B)). The results demonstrate the effectiveness of THBA in delaying fibrin gelation when its concentration is above 100 nM.

To reverse the inhibition and initiate rapid fibrin gelation, the DNA triggers were added to the mixture containing THBA-inhibited thrombin and fibrinogen at different molar ratio to THBA, and the absorbance at 350 nm was monitored again *via* the turbidity assay. It was observed that the addition of DNA trigger at 0.5 : 1 was able to alleviate the inhibition caused by THBA. The alleviation effect of DNA triggers continued to increase until reached the molar ratios of 5 : 1, indicating that the alleviation effect reached saturation when DNA trigger administration was at molar ratio of 5 : 1 (Fig. 3(C)). When we analyzed the time required to reach 50% maximum absorbance, it was found that the administration of DNA trigger in a molar ratio of 0.5 : 1 reduced the time by 46%. For DNA trigger in the range of molar ratio from 1 : 1 to 10 : 1, the time was reduced by an average of 66% (Fig. 3(D)). This significant decrease showed that the DNA trigger could reverse the THBA-mediated thrombin inhibition to accelerate fibrin gelation. Moreover, Fig. 3(C) confirmed that the exosite I-targeting strands were dissociated from thrombin since the addition of DNA trigger molecule had increased the fibrin gelation as indicated by an increase in absorbance value measured at 350 nm, and dissociations of exosite I-targeting strands were required for fibrin to access the catalytic site of thrombin.

The inhibition of fibrin gelation and the subsequent initiation of gelation regulated by DNA trigger was also examined. The vials containing gel solutions under different treatments (without inhibition, inhibition caused by single stranded bispecific aptamer, THBA, and THBA-inhibited with DNA trigger) were tilted to determine fibrin gelation. At 120 s, the fibrin hydrogel inhibited by single stranded bispecific aptamer (SBA) and THBA exhibited solution form (Fig. 3(E)). The result shows that THBA resulted in similar levels of inhibition as SBA. For THBA-inhibited fibrin hydrogel added with DNA trigger, the gel had already formed at 120 s. The efficiency of DNA triggers to recover fibrin gelation from SBA inhibition was studied for comparison. As shown in Fig. S4 (ESI†), the addition of DNA triggers, either targeting the same regions on THBA or 15-mer and 29-mer, resulted in poor initiation of gelation in the fibrin hydrogel inhibited by SBA. This observation indicates the lower efficiency of SBA in allowing the inhibition to be reversed by DNA trigger. Collectively, the data shows that THBA can exert effective inhibition while allowing the inhibition to be easily reversed by the DNA trigger.

The rheological analysis also showed inhibition in fibrin gelation as the storage (*G*′) and loss modulus (*G*′′) of THBA-inhibited fibrin hydrogel was much lower in the beginning; however, the uninhibited fibrin hydrogel and DNA triggered fibrin hydrogel after inhibition exhibited higher initial storage and loss modulus, showing more partially formed hydrogel network (Fig. S5, ESI†). This rheology experiment aims to mimic the scenario of clinically used fibrin sealants since commercialized fibrin sealants such as *TISSEEL* is mainly composed of human fibrinogen and thrombin in CaCl_2_.^36^ The *G*′ of THBA-inhibited thrombin–fibrinogen mixture induced by DNA trigger rapidly increased when compared to the mixture without DNA trigger. The change in *G*′ in THBA-inhibited fibrin hydrogel initiated by DNA highly resembles the one in fibrin hydrogel without THBA inhibition. From a translational perspective, clinically available fibrin sealants such as *TISSEEL* polymerize within seconds upon thrombin–fibrinogen mixing, which is not feasible for minimally invasive delivery over long distances. The system proposed in this study may allow the co-delivery of thrombin and fibrinogen over longer distances without fibrin hydrogel formation inside devices such as a catheter.

### Examination of fibrin polymerization at microscopic level under THBA inhibition and DNA trigger initiation

**Fig. 4:**
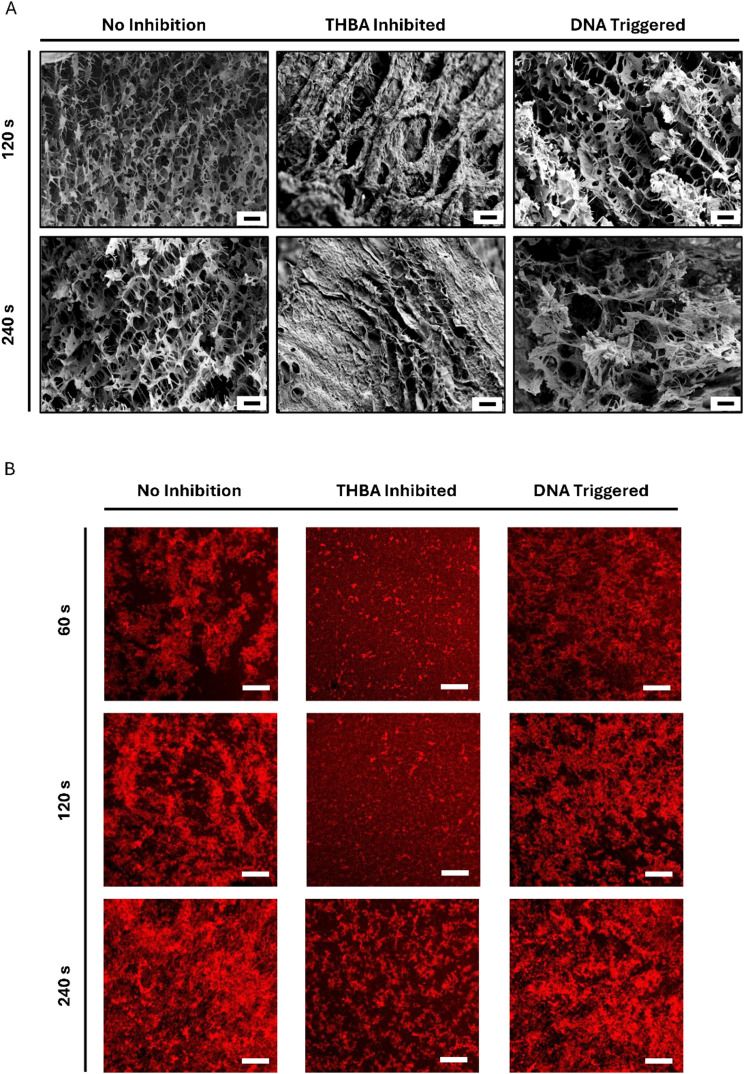
Microscopic analysis of fibrin prepared under different conditions. (A) Scanning electron microscopy (SEM) images. The state of fibrin polymerization process was fixed at 120 and 240 s by rapidly freezing in liquid nitrogen. Scale bar = 10 μm. (B) Confocal microscopy images. The coverslip was overlaid on top of the fibrin hydrogels, and photos were taken at 60, 120, and 240 s. Scale bar = 200 μm. The THBA concentration was 250 nM. The DNA trigger-to-THBA molar ratio was 10 : 1.

Fibrin gelation started with thrombin cleavage of fibrinopeptides to generate fibrin monomers, followed by fibrin polymerization in which the fibrin monomers aggregate and polymerize.^37,38^ Scanning electron microscopy (SEM) was used to analyze the microstructure during fibrin polymerization. In the fibrin pre-gel solution with thrombin inhibited by THBA, no apparent fibrous structure was observed from the SEM images at both 120 s and 240 s, indicating the inhibition of thrombin was successful and fast fibrin polymerization did not occur. In the fibrin pre-gel solution with THBA-inhibited thrombin and DNA trigger, a crosslinked fibrous structure that highly resembles the structure in fibrin hydrogel without inhibition was already observed at 120 s (Fig. 4(A)). This indicates that although thrombin was inhibited by THBA, the addition of DNA trigger effectively restored the polymerization of fibrin. This was due to the disruption in hybridized formation of THBA, which subsequently caused the 15-mer strand to dissociate from thrombin and exposed thrombin exosite I to fibrinogen. The extent of fibrin polymerization was also evaluated using confocal microscopy. Fibrin polymerization and aggregation was found at 60 s in fibrin hydrogel without aptamer inhibition, and the aggregation became denser as time progresses. For the fibrin hydrogel with THBA inhibition, no large clusters of fibrin aggregation were observed for up to 120 s and only a small amount of fibrin aggregation appeared at 240 s. The addition of DNA trigger in THBA inhibited fibrin hydrogel resulted in the formation of some fibrin aggregation at 60 s and the degree of fibrin aggregation increased with time (Fig. 4(B)). These data collectively showed that the THBA was effective in inhibiting fibrin polymerization and the DNA trigger treatment resulted in initiation of fibrin polymerization in which the extent of polymerization was similar to uninhibited fibrin hydrogel.

### Injectability of THBA-inhibited fibrin hydrogel

The injectability of THBA-inhibited fibrin hydrogel was tested by injection through a 23G needle. To compare the injectability of fibrin hydrogel inhibited by THBA with SBA, the fibrin hydrogels were injected into a vial containing DPBS. It was found that the fibrin hydrogel without aptamer inhibition was uninjectable at 240 s, whereas both SBA and THBA inhibited fibrin exhibited pre-gel solution form and were injectable (Fig. S6A, ESI†). The gelation triggered by the complementary DNA was also tested in its injectability. The THBA-inhibited fibrin hydrogel was extruded in the form of low-viscosity solution whereas the DNA trigger-added THBA-inhibited fibrin hydrogel was extruded in the form of a highly viscous material at 120 s (Fig. S6B, ESI†). The results collectively proved the injectability of the fibrin hydrogel with THBA inhibition, and the DNA trigger treatment induced gelation.

### Biocompatibility of THBA–DNA modulated fibrin hydrogel

**Fig. 5:**
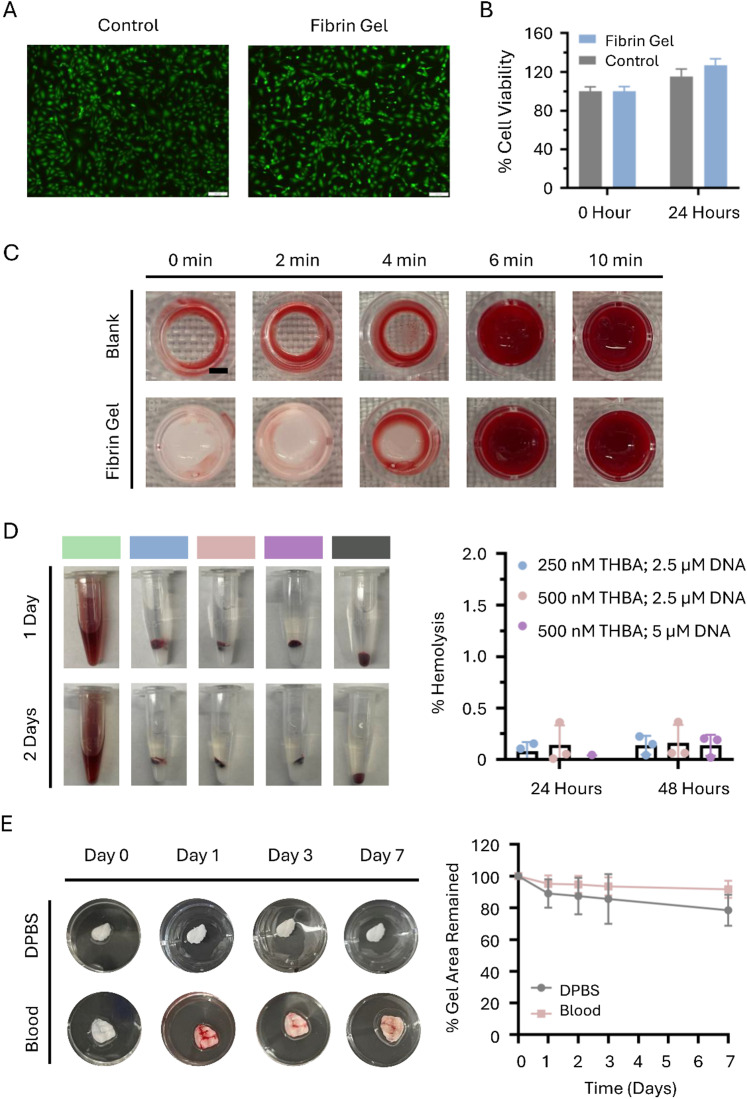
Biocompatibility of THBA-inhibited DNA-triggered fibrin hydrogel. (A) Viability staining of HUVECs co-cultured with the THBA–DNA modulated fibrin hydrogel for 24 hours. Green = live cells; red = dead cells. Scale bar = 200 μm. (B) Cell viability of HUVECs cultured with release media of THBA–DNA modulated fibrin hydrogel quantified using MTS assay. (C) Clotting behavior of blood incubated with THBA–DNA modulated fibrin hydrogel. The aptamer concentration was 250 nM. The DNA trigger-to-THBA molar ratio was 10 : 1. Scale bar = 2 mm. (D) Hemolysis analysis of red blood cells (RBCs) incubated with THBA–DNA modulated fibrin hydrogel. Left: Photographs of RBCs incubated with fibrin hydrogels containing different THBA and DNA concentrations. Green = Triton X-100; blue = 250 nM of THBA and 2.5 μM of DNA trigger; pink = 500 nM of THBA and 2.5 μM of DNA trigger; purple = 500 nM THBA and 5 μM of DNA trigger; gray = RBC-only (negative control). Right: Percent hemolysis of RBCs incubated with different fibrin hydrogels. (E) Degradation analysis of THBA–DNA modulated fibrin hydrogel. The aptamer concentration was 250 nM. The DNA trigger-to-THBA molar ratio was 10 : 1.

The cytocompatibility of fibrin hydrogel with THBA inhibition followed by gelation triggered by DNA was analyzed by viability staining after transwell co-culture with HUVECs. The cytotoxicity of the release media from the fibrin hydrogel was assessed by culturing HUVECs with the release media, followed by MTS assay to evaluate cell viability. The treatment without THBA–DNA modulated fibrin hydrogel was served as the control. The viability staining showed similar levels of live cells between the no treatment control and the THBA–DNA modulated fibrin hydrogel (Fig. 5(A)). The results of the MTS assay were consistent with those from the viability staining (Fig. 5(B)). The data demonstrated that the fibrin hydrogel formed following THBA inhibition and DNA triggering was non-cytotoxic, and that the released aptamers and DNAs also exhibited no cytotoxic effects.

Since the injectable hydrogel will be in contact with the blood, hemocompatibility evaluations including clotting and hemolysis were performed. From the clotting assay, we found that the fibrin hydrogel, first inhibited by THBA and then polymerized upon DNA triggering, did not significantly change the clotting behavior of the blood (Fig. 5(C)). To examine whether THBA–DNA modulated fibrin hydrogel caused hemolysis, fibrin hydrogels treated with different concentrations of THBA and DNA trigger were incubated with red blood cells (RBCs) for 24 and 48 hours. The results show that regardless of the THBA and DNA concentrations tested, the hemolysis resulted from the incubation with fibrin hydrogel was less than 0.5%, which is negligible (Fig. 5(D)). The results were expected since nucleic acid aptamers offer the advantages of minimal toxicity and high biocompatibility. Together, these data demonstrated high hemocompatibility of the THBA-inhibited hydrogel triggered by complementary DNA.

The stability of the fibrin hydrogel in the blood was also analyzed. The THBA–DNA modulated fibrin hydrogel did not show differences in gel degradation in the blood compared to DPBS (Fig. 5(E)), and there were 80% of the fibrin hydrogel remaining for up to one week. This observation indicated that the THBA inhibition did not cause the hydrogel to be more prone to degradation in the blood.

### Assessment of THBA–DNA modulated fibrin hydrogel in embolization of *in vitro* aneurysm model

**Fig. 6:**
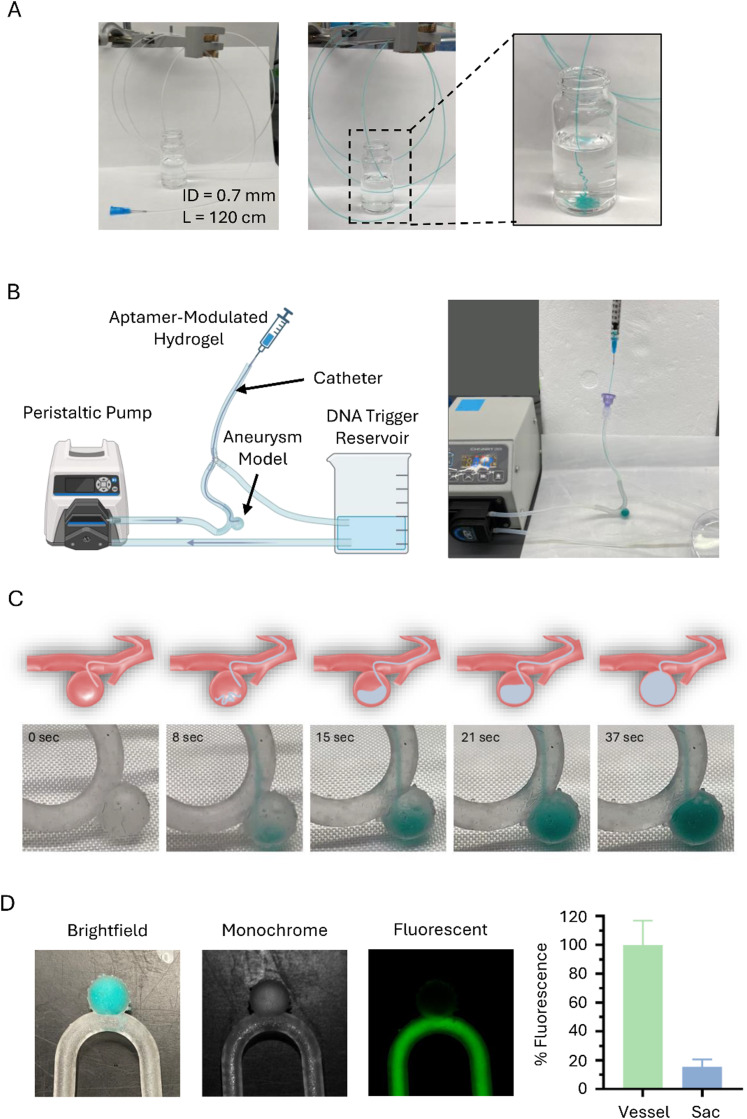
Embolization of *in vitro* aneurysm model. (A) Injectability of THBA–DNA modulated fibrin hydrogel over catheter with inner diameter of 0.7 mm and total length of 120 cm at 240 s. (B) Schematic illustration of *in vitro* aneurysm system (left) and photograph of the system (right). (C) The process of gel delivery *via* catheter. Flow rate = 5 mL min^−1^. (D) Evaluation of embolic ability of THBA–DNA modulated fibrin hydrogel by perfusing FITC-dextran solution through. Right-most: quantification of mean fluorescence intensity from FITC in vessel *vs.* sac regions. The aptamer concentration was 250 nM. The DNA trigger-to-THBA molar ratio was 10 : 1.

The feasibility of delivering the THBA-inhibited fibrin hydrogel *via* catheter was assessed. A catheter with an inner diameter of 0.7 mm and total length of 120 cm was used. The THBA-inhibited fibrin hydrogel was shown to be injectable through the catheter over the distance of 120 cm (Fig. 6(A)), demonstrating its suitability for catheter-delivered injectable hydrogel applications, enabling minimally invasive delivery of this embolic and hemostatic biomaterial. The applicability of the injectable THBA-inhibited fibrin hydrogel in embolization of aneurysm was evaluated in an *in vitro* saccular aneurysm model as shown in Fig. 6(B). The system was first perfused with DNA trigger solution pumping from the reservoir at 5 mL min^−1^ to simulate the systemic circulation of DNA trigger in the body. Following that, the catheter was inserted through the tubing closed by a Tuohy valve and into the aneurysm sac. The injectable THBA-inhibited fibrin hydrogel was then delivered through the catheter to gradually fill the site of aneurysm and mixed with the DNA trigger to form a gel (Fig. 6(C)). The efficiency of DNA-triggered THBA-inhibited fibrin hydrogel in occluding aneurysm site was evaluated by perfusing a fluorophore solution through the perfusion system. The fluorescence detected in the sac was minimal and the average percent fluorescence intensity in the aneurysm sac was about 10%, demonstrating the success of the fibrin hydrogel to fill and block the aneurysm sac (Fig. 6(D)).

### Evaluation of THBA–DNA modulated fibrin hydrogel in endovascular embolization

**Fig. 7:**
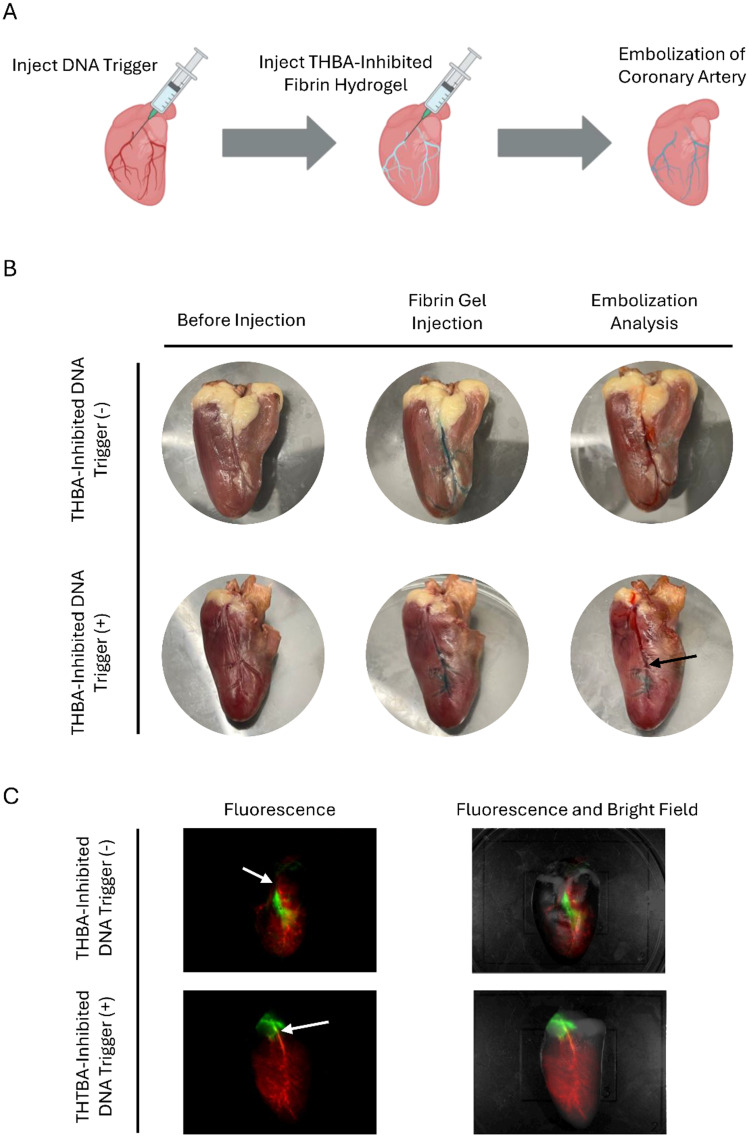
*In vitro* evaluation of endovascular embolization. (A) Scheme showing endovascular embolization in a heart model. (B) Embolization of artery by THBA-inhibited fibrin hydrogels with or without DNA trigger demonstrated in colored food dye. Black arrow: point of embolization. (C) Embolization by THBA-inhibited fibrin hydrogels with or without DNA trigger demonstrated in fluorescently labeled fibrin hydrogel and FITC-dextran solution. White arrow: point of fibrin hydrogel injection. The aptamer concentration was 250 nM. The DNA trigger-to-THBA molar ratio was 10 : 1.

THBA-inhibited fibrin hydrogel was also tested for its applicability in endovascular embolization using a chicken coronary artery as the model (Fig. 7(A)). For the test group with DNA trigger, the DNA trigger was first injected into the coronary artery whereas the group without DNA trigger was injected with saline solution. The THBA-inhibited hydrogel was subsequently injected. The THBA-inhibited hydrogel, as shown in blue, was able to be injected smoothly and travelled into thinner vasculature of the chicken heart close to the apex. It showed the feasibility of using THBA-inhibited fibrin hydrogel as an injectable hydrogel to be delivered into the microvasculature, which may be difficult to reach by using other injectable hydrogels due to their fast gelation kinetics. To evaluate endovascular embolization, a saline solution colored with red food dye was injected into the coronary artery. In the hydrogel without DNA trigger, the red saline solution was able to displace the blue pre-gel solution, indicating that the gel did not form, and the vasculature was not occluded. For the sample injected with DNA triggers followed by THBA-inhibited hydrogel, the red saline solution was only able to travel to the midway of the vessel and started backflowing (Fig. 7(B)). This observation was due to the formation of fibrin hydrogel initiated by the DNA trigger previously injected. To further demonstrate the effectiveness of endovascular embolization, the fibrin gel was visualized by using Cy5-labeled fibrinogen, and the saline solution was visualized by dissolving FITC-dextran. For THBA-inhibited fibrin hydrogel without DNA trigger, it was apparent that the fibrin hydrogel did not form, and the injection of FITC-dextran saline solution was able to travel towards the apex of the chicken heart. The THBA-inhibited fibrin hydrogel with DNA trigger showed effective blocking of the vasculature and the FITC-dextran saline solution injected was not able to travel along the occluded vasculature and resulted in a back flow of FITC-dextran solution towards the base (top) of the heart (Fig. 7(C)). Collectively, these experiments demonstrated the feasibility of using THBA–DNA modulated fibrin hydrogel as an endovascular embolic agent.

The fibrin hydrogels possess high biocompatibility, however, their applications in minimally invasive delivery over catheter were limited by their rapid gelation rate. To enable their injectability and delivery through a long catheter, bispecific aptamers with high affinity were used to inhibit the gelation to allow the fibrin hydrogel to be delivered in a solution form. Moreover, to ensure both injectability and rapid gelation, a DNA trigger was designed to dissociate the bispecific aptamer from thrombin and neutralize the hybrid bispecific aptamer for releasing thrombin and inducing rapid fibrin gelation. The DNA trigger can be systematically delivered through methods such as intravenous injection, which is also minimally invasive. Upon contact with DNA trigger, the locally injected fibrin in the form of solution will start to form a hydrogel *in situ*. This is especially useful for embolization of micro-level cavities or vessels, since the hydrogel and the trigger both have excellent flowability.

One study reported the use of a polyethylene glycol (PEG)-based injectable hydrogel, formed *via* alkyne-azide cycloaddition, as an embolic agent.^39^ The hydrogel exhibited a gelation time of 5 minutes and was effective in embolizing the auricular central artery in rabbit models. Similarly, another study evaluated a self-healing hydrogel composed of chitosan and PEG for renal artery embolization in rats.^40^ This hydrogel gelled within 3 minutes and successfully achieved embolization. By collectively comparing the gelation time of our gelation system with the results discussed above, the aptamer-modulated injectable hydrogel system has potential in translational endovascular embolization.

Aptamer-modulated injectable hydrogel triggered by the complementary DNAs could potentially face some clinical translation barriers such as *in vivo* DNA stability and degradation. The rapid renal clearance of DNA oligonucleotides is also an issue in nucleic acid-based therapies. However, DNA stability can be improved by chemical modifications such as phosphorothioate backbone substitutions against nuclease degradation.^41^ Conjugating DNAs to polymers such as polyethylene glycol can improve the half-life of the DNA in circulation.^42^

## Conclusion

A hybrid bispecific aptamer has been developed to inhibit thrombin-mediated conversion of fibrinogen to fibrin, enabling the injectability of fibrin hydrogels through needles and catheters. This pre-gelation system can then be triggered to form stable and biocompatible hydrogels using specifically designed DNA molecules that disrupt the aptamer-mediated thrombin inhibition. This study thus presents a promising *in situ* injectable hydrogel platform regulated by aptamers and DNA triggers. While traditional injectable hydrogels eliminate the need for invasive implantation, they must also satisfy controllable gelation kinetics. The hydrogel system described here meets this challenge through innovations in both biomolecular design and material engineering. Furthermore, the capability to introduce blood-borne DNA triggers paves the way for next-generation biomaterials whose components can remain separated until selectively activated *in situ*. This mechanism is applicable not only within the vascular system but also in diverse environments such as living cells. As a result, these systems hold significant potential for a wide range of applications beyond vascular embolization and bleeding control.

## Materials and methods

### Materials

Fibrinogen and thrombin from human plasma were obtained from Sigma-Aldrich. Biotinylated thrombin and the live/dead viability/cytotoxicity Kit were supplied by Thermo Fisher Scientific. The CellTiter 96® Aqueous One Solution Cell Proliferation Assay was acquired from Promega. All aptamers and complementary DNA (cDNA) were obtained from Integrated DNA Technologies. Streptavidin-coated polystyrene microparticles (0.5% w/v, 5.0 μm) were purchased from Spherotech. Needles and syringes were acquired from Fisher Scientific. DBCO-PEG_4_-NHS ester and Cy5-azide were acquired from Click Chemistry Tools. Human umbilical vein endothelial cells (HUVECs) were obtained from ATCC, while citrate-phosphate-dextrose (CPD)-treated porcine whole blood was purchased from Lampire Biological Laboratories.

### Design and formation of hybrid bispecific aptamer and DNA trigger

To yield a hybrid bispecific aptamer that effectively inhibits thrombin, a 5T spacer and 12-nucleotide sticky end were added to the 3′ end of each 15-mer and 29-mer of thrombin. The sticky ends on 15-mer are complementary to the sticky ends on 29-mer. For the hybrid bispecific aptamer with improved DNA triggered dissociation, a toehold sequence was designed at the 3′ end of 29-mer. The hybrid bispecific aptamer was formed through the hybridization of complementary sticky region on 15-mer and 29-mer by heating the aptamer mixtures (at molar ratio of 1 : 1) to 95 °C and cooled down on ice. The formation of hybrid bispecific aptamer was confirmed by running gel electrophoresis with 12% polyacrylamide gel. The DNA trigger was designed to complementary hybridize with the last five bases of 29-mer plus the 5T spacer, sticky region, and toehold sequence. The single stranded bispecific aptamer used in this study as a control is composed of 15-mer and 29-mer connected by 22T linker.

### Binding of hybrid bispecific aptamer–thrombin and nucleic acid trigger mediated release

The binding of hybrid bispecific aptamer to thrombin was assessed using microparticle flow cytometry method as reported in previous work.^27^ Briefly, biotinylated thrombin was incubated with streptavidin-functionalized polystyrene microparticles (0.5% w/v, 5.0 μm) to obtain thrombin-microparticles. Different concentrations of hybrid bispecific aptamer with carboxyfluorescein (FAM) labeled on the 5′ end of 15-mer was incubated with thrombin-microparticles and washed with DPBS before analyzing with flow cytometry (Attune NxT Flow Cytometer, Thermo Fisher Scientific). The data obtained from flow cytometry was processed using FlowJo v10.8.1. The mean fluorescence intensity (MFI) was plotted against the aptamer concentration and fitted to generate a binding curve. GraphPad PRISM 8 was used to perform curve fitting to determine the dissociation constant. To observe the DNA triggered dissociation of hybrid bispecific aptamer from thrombin, the DNA triggers were incubated with aptamer-bound thrombin microparticles and washed with DPBS prior to being analyzed with flow cytometry. The percentage of aptamer dissociation was calculated as follows:
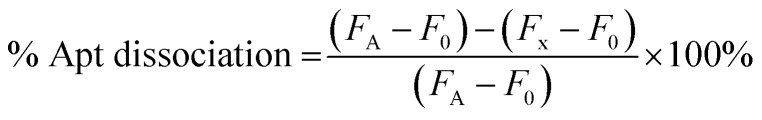
*F*_A_ is the MFI of aptamer-particles without treatment of DNA trigger, *F*_0_ is the MFI of blank particles, and *F*_x_ is the MFI of aptamer-particles treated with DNA triggers at different molar ratios.

### Synthesis of toehold hybrid bispecific aptamer (THBA)-DNA modulated fibrin hydrogel

Fibrin hydrogel was formed by mixing equal volumes of fibrinogen and thrombin. Unless specified otherwise, 25 mg mL^−1^ of fibrinogen and 2.5 U mL^−1^ of thrombin in 10 mM CaCl_2_ were used for fibrin hydrogels synthesis in this study. To inhibit thrombin-catalyzed fibrin hydrogel formation, aptamers at pre-determined concentrations were incubated with thrombin for 30 minutes before mixing with fibrinogen. For DNA trigger mediated gelation, the DNA triggers of the aptamers were added at different trigger-to-aptamer molar ratios at 30 seconds after the fibrinogen and aptamer-inhibited thrombin were mixed.

### Turbidity assay for monitoring fibrin hydrogel gelation (inhibition and activation)

Turbidity assays were conducted according to the method previously reported.^43^ Briefly, the fibrinogen was mixed with thrombin (final volume of 100 μL) and added into 96-well plate, and the absorbances at 350 nm (A_350_) over time were measured using a plate reader (Tecan Infinite M200 Pro). To examine the effect of DNA trigger addition on the turbidity change in fibrin hydrogel, the DNA triggers of different molar ratio to THBAs were added at 30 s after thrombin fibrinogen mixing. In this study, the absorbance at 50% of the maximum turbidity was determined using an uninhibited fibrin hydrogel, and the time required for solutions under different treatments to reach that absorbance value was determined from the *A*_350_*versus* time plot.

### Analysis of fibrin hydrogel formation by rheology

The rheological behavior of fibrin hydrogel was analyzed using a rheometer Discovery HR 20 (TA Instruments). Fibrin hydrogels (400 μL) with different treatments were prepared and loaded onto the lower Peltier plate that was held at 37 °C. An upper stainless-steel plate with a diameter of 40 mm and 1° angle was used. The measurement gap between the plates was set to 23 μm and the time sweep test was performed for 5 minutes at 1.0 Hz and 0.1% strain.

### Analysis of fibrin hydrogel microstructure

Fibrin hydrogels, with or without aptamer-mediated inhibition, were prepared as previously described. DNA triggers were introduced at 30 s after mixing fibrinogen with aptamer-inhibited thrombin. At 120 s and 240 s post-mixing, the reaction was rapidly quenched by freezing in liquid nitrogen. The frozen hydrogels were then lyophilized, coated with iridium, and imaged using a scanning electron microscope (SIGMA VP-FESEM, Zeiss).

### Analysis of fibrin polymer formation by confocal microscopy

Cy5-labeled fibrinogen was synthesized using copper-free click chemistry with Dibenzocyclooctyne (DBCO). To begin, DBCO-modified fibrinogen was prepared by reacting 50 mg of fibrinogen with DBCO-PEG_4_-NHS ester at a 1 : 50 molar ratio in 50 mM NaHCO_3_ buffer. The reaction proceeded for 3 hours at room temperature on a shaker set to 100 rpm. Following the reaction, unreacted DBCO was removed through repeated washing with 1× PBS and centrifugation using a 100 kDa centrifugal filter at 14 000 × *g* for 10 minutes. The presence of residual DBCO in the filtrates was assessed by monitoring absorbance at 310 nm using a Nanodrop 2000c Spectrophotometer. Washing and centrifugation were repeated until no unreacted DBCO was detected.

The purified fibrinogen-DBCO was then reacted with Cy5-azide at a 1 : 1 molar ratio in 0.05 M Tris–HCl buffer (pH 8.0) and incubated on a shaker at 100 rpm for 4 hours at room temperature. Unreacted Cy5-azide was removed by additional rounds of PBS washing and centrifugation under the same conditions. Filtrates were analyzed for fluorescence at 665 nm using a Nanodrop 3300 Fluorospectrometer to confirm removal of excess dye.

The resulting Cy5-labeled fibrinogen was combined with thrombin—with or without prior aptamer inhibition—as described above. Ten microliters of the mixture were pipetted onto a microscope glass coverslip and overlaid with a second coverslip. Images were captured at specified time points.

### Assessment of fibrin hydrogel injectability

The injectability of fibrin hydrogels (200 μL) under different treatments were evaluated *via* injection through 25 G needles and catheters with inner diameters of 0.7 mm and total lengths of 120 cm.

### Evaluation of biocompatibility of THBA–DNA modulated fibrin hydrogel

The cytocompatibility of fibrin hydrogels treated with THBA and DNA trigger were evaluated by analyzing the viability of HUVECs co-cultured with fibrin hydrogel and cultured with the release media of fibrin hydrogel. The cell viability of HUVEC was assessed by viability staining (LIVE/DEAD™ Viability/Cytotoxicity Kit, Invitrogen) and MTS assay (CellTiter 96® Aqueous One Solution Cell Proliferation Assay, Promega). Briefly, 50 000 and 5000 HUVECs were seeded in each well of 24-well and 96-well plate, respectively. The HUVECs were cultured for 24 hours at 37 °C with 5% CO_2_ in M200 supplemented with 2% low serum growth supplement (LSGS). In cell viability staining assay, the fibrin hydrogels were co-cultured with HUVECs for 24 hours using Transwell culture insert (PET membrane, 8.0 μm pores) in 24-well plate. To obtain the release media for MTS assay, the fibrin hydrogel (100 μL) was incubated in complete growth medium for 24 hours. The release media was subsequently incubated with HUVECs in 96-well plate for 24 hours, followed by MTS assay.

The hemocompatibility of fibrin hydrogel was evaluated in clotting and hemolysis assays using CPD-treated porcine whole blood. For clotting assay, 100 μL of fibrin hydrogels were synthesized in 96-well plate, and the CPD-treated blood was mixed with 0.1 M of CaCl_2_ solution and added to empty wells (control) or wells containing fibrin hydrogels. At pre-determined time points, each well was rinsed with DPBS, and the clotting behavior at each time point was observed.

For hemolysis assay, the CPD-treated blood was centrifuged at 500 × *g* for 10 minutes and the supernatant was removed to obtain red blood cell (RBC) pellet. The RBC pellet was resuspended in DPBS at volume ratio of 1 : 7. Subsequently, 200 μL of the RBC suspension was incubated with DPBS (negative control), DPBS + fibrin hydrogel (sample), and 2% v/v Triton X-100 in DPBS (positive control) at 37 °C. At pre-determined time points, the mixtures were centrifuged at 800 × *g* for 15 minutes. The supernatant was transferred to 96-well plate and the absorbance (Abs) at 545 nm was measured. The percentage of hemolysis was calculated as follows:
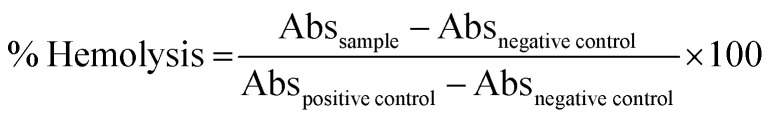


The analysis of fibrin hydrogel degradation in porcine whole blood was studied by incubating pre-formed fibrin hydrogel (100 μL) in DPBS and whole blood at 37 °C. At pre-determined time points, the fibrin hydrogels were rinsed with DPBS, and the area of the hydrogel was quantified using ImageJ to analyze the hydrogel degradation.

### Evaluation of fibrin hydrogel in embolization of *in vitro* aneurysm model and chicken heart model

For embolization of an *in vitro* aneurysm model, a saccular aneurysm replica^44^ with some modifications was 3D-printed with Elastic 50A resin using Form3 SLA printer (FormLabs, MA). The inner diameter of the sac and branched vessel was 8 mm and 3 mm, respectively. The aneurysm replica was connected to a peristaltic pump and fluid reservoir *via* silicone tubes with a diameter of 2.38 mm to form a fluidic circulation system. The flow velocity of DPBS or DNA trigger solution was 5 mL min^−1^. To deliver fibrin hydrogel *via* catheter, the catheter (ID = 0.7 mm) was connected to 1 mL syringe loaded with 300 μL of fibrin hydrogel was inserted through a Tuohy valve and reached the aneurysm site. To confirm the embolization efficiency of aneurysm sac by the fibrin hydrogel delivered through catheter, a solution with FITC-dextran (*M*_W_ = 10 000) was pumped into the system and the mean fluorescence intensity in the sac and the vessels were compared. The fluorescence in the sac was calculated and presented in percentage of fluorescence with respect to the vessel in which the fluorescence in the vessel was set to 100%.

For the *in vitro* embolization of chicken heart vasculature, the chicken heart was obtained from local grocery stores. The DNA trigger was first injected into the coronary arteries of the chicken heart, followed by injections of fibrin hydrogels with blue food dye for visualization through a 30 G needle. At 5 minutes after fibrin gelation inside the coronary arteries, a saline solution with red food dye was injected into the coronary arteries to visualize the effect of embolization. The same procedure was repeated using Cy5-labeled fibrinogen for visualization of fibrin hydrogel and FITC-dextran solution as the perfusion solution to confirm the success of embolization.

### Statistical analysis

Statistical significance among samples was assessed using one-way analysis of variance (ANOVA). Dunnett's *post-hoc* test was subsequently applied to compare each experimental group to the control group. All the data were reported as mean ± standard error of the mean (SEM). All statistical analyses were conducted using GraphPad PRISM 8.

## Conflicts of interest

There are no conflicts to declare.

## Supplementary Material

NH-010-D5NH00314H-s001

## Data Availability

The data supporting the findings of this study are available within the article and its ESI† files. Additional datasets generated and analyzed during the current study are available from the corresponding author upon reasonable request.

## References

[cit1] Yang W. J., Zhou P., Liang L., Cao Y., Qiao J., Li X., Teng Z., Wang L. (2018). ACS Appl. Mater. Interfaces.

[cit2] Liu N., Wu S., Tian X., Li X. (2022). J. Mater. Chem. B.

[cit3] Hu S., Dai Y., Xin L., Zheng X., Ye Z., Zhang S., Ma L. (2024). Acta Biomater..

[cit4] Dong R., Zhao X., Guo B., Ma P. X. (2016). ACS Appl. Mater. Interfaces.

[cit5] Xu Q., A S., Gao Y., Guo L., Creagh-Flynn J., Zhou D., Greiser U., Dong Y., Wang F., Tai H., Liu W., Wang W., Wang W. (2018). Acta Biomater..

[cit6] Zhou L., Dai C., Fan L., Jiang Y., Liu C., Zhou Z., Guan P., Tian Y., Xing J., Li X., Luo Y., Yu P., Ning C., Tan G. (2021). Adv. Funct. Mater..

[cit7] Zhao P., Yang P., Zhou W., Liu H., Jin X., Zhu X. (2023). Adv. Healthcare Mater..

[cit8] Fujiwara S., Yoshizaki Y., Kuzuya A., Ohya Y. (2021). Acta Biomater..

[cit9] Shi J., Wang D., Wang H., Yang X., Gu S., Wang Y., Chen Z., Chen Y., Gao J., Yu L., Ding J. (2022). Acta Biomater..

[cit10] Sun L., Ouyang J., She Z., Li R., Zeng F., Yao Z., Wu S. (2024). Adv. Healthcare Mater..

[cit11] Liu M., Sun Y., Zhou Y., Chen Y., He C., Zhao Q., Yan L., He J., Guo J., Liu K., Li Y., Lin J., Liao W., Shan H., Peng X. (2025). Adv. Funct. Mater..

[cit12] Fan L., Duan M., Xie Z., Pan K., Wang X., Sun X., Wang Q., Rao W., Liu J. (2020). Small.

[cit13] Xie R., Chen Y.-C., Zhao Y., Yodsanit N., Wang Y., Yamamoto N., Yamanouchi D., Gong S. (2021). ACS Appl. Mater. Interfaces.

[cit14] Dutta K., Das R., Ling J., Monibas R. M., Carballo-Jane E., Kekec A., Feng D. D., Lin S., Mu J., Saklatvala R., Thayumanavan S., Liang Y. (2020). ACS Omega.

[cit15] Geng S., Zhao H., Zhan G., Zhao Y., Yang X. (2020). ACS Appl. Mater. Interfaces.

[cit16] Li L., Gu J., Zhang J., Xie Z., Lu Y., Shen L., Dong Q., Wang Y. (2015). ACS Appl. Mater. Interfaces.

[cit17] Wang Y., Wang S., Hu W., Kong S., Su F., Liu F., Li S. (2023). J. Pharm. Sci..

[cit18] Li C., Wang J., Niu Y., Zhang H., Ouyang H., Zhang G., Fu Y. (2023). ACS Appl. Mater. Interfaces.

[cit19] Dimatteo R., Darling N. J., Segura T. (2018). Adv. Drug Delivery Rev..

[cit20] Mandell S. P., Gibran N. S. (2014). Expert Opin. Biol. Ther..

[cit21] Holcomb J. (1998). Arch. Surg..

[cit22] Larson M. J. (1995). Arch. Surg..

[cit23] Shekarriz B., Stoller M. L. (2002). J. Urol..

[cit24] Spotnitz W. D. (2001). Am. J. Surg..

[cit25] Huntington J. A. (2005). J. Thromb. Haemostasis.

[cit26] Verhamme I. M., Olson S. T., Tollefsen D. M., Bock P. E. (2002). J. Biol. Chem..

[cit27] Wen C., Lee K., Wang Y., Wang X., Wang Y. (2024). Langmuir.

[cit28] Kim Y., Cao Z., Tan W. (2008). Proc. Natl. Acad. Sci. U. S. A..

[cit29] Bock L. C., Griffin L. C., Latham J. A., Vermaas E. H., Toole J. J. (1992). Nature.

[cit30] Tasset D. M., Kubik M. F., Steiner W. (1997). J. Mol. Biol..

[cit31] Manning G. S. (2006). Biophys. J..

[cit32] Paoletti F., El-Sagheer A. H., Allard J., Brown T., Dushek O., Esashi F. (2020). EMBO J..

[cit33] Hasegawa H., Taira K., Sode K., Ikebukuro K. (2008). Sensors.

[cit34] Zhang D. Y., Winfree E. (2009). J. Am. Chem. Soc..

[cit35] Zadeh J. N., Steenberg C. D., Bois J. S., Wolfe B. R., Pierce M. B., Khan A. R., Dirks R. M., Pierce N. A. (2011). J. Comput. Chem..

[cit36] Spotnitz W. D., Burks S. (2008). Transfusion.

[cit37] Weisel J. W., Litvinov R. I. (2013). Blood.

[cit38] Stamboroski S., Joshi A., Noeske P. M., Köppen S., Brüggemann D. (2021). Macromol. Biosci..

[cit39] Su X., Bu L., Dong H., Fu S., Zhuo R., Zhong Z. (2016). RSC Adv..

[cit40] Zhou X., Li Y., Chen S., Fu Y., Wang S., Li G., Tao L., Wei Y., Wang X., Liang J. F. (2018). Colloids Surf., B.

[cit41] Crooke S. T., Vickers T. A., Liang X. (2020). Nucleic Acids Res..

[cit42] Harris J. M., Chess R. B. (2003). Nat. Rev. Drug Discovery.

[cit43] Soon A. S. C., Lee C. S., Barker T. H. (2011). Biomaterials.

[cit44] Goetz A., Jeken-Rico P., Chau Y., Sédat J., Larcher A., Hachem E. (2024). Bioengineering.

