# Supplementary material for: DNA-triggered activation of aptamer-neutralized enzyme for in situ formation of injectable hydrogel
Source: Nanoscale Horiz. 2025 Jun 16;10(8):1703–16. doi: 10.1039/d5nh00314h (PMC12169211; doi:10.1039/d5nh00314h)
Supplement: NH-010-D5NH00314H-s001 [file NH-010-D5NH00314H-s001.pdf]

Supplementary Information (SI) for Nanoscale Horizons.
This journal is © The Royal Society of Chemistry 2025

DNA-Triggered Activation of Aptamer-Neutralized Enzyme for In Situ Formation of Injectable Hydrogel

*Connie Wen, Yixun Wang, Kyungsene Lee, Xuelin Wang, Yong Wang**

Department of Biomedical Engineering, The Pennsylvania State University, University Park, PA 16802, USA.

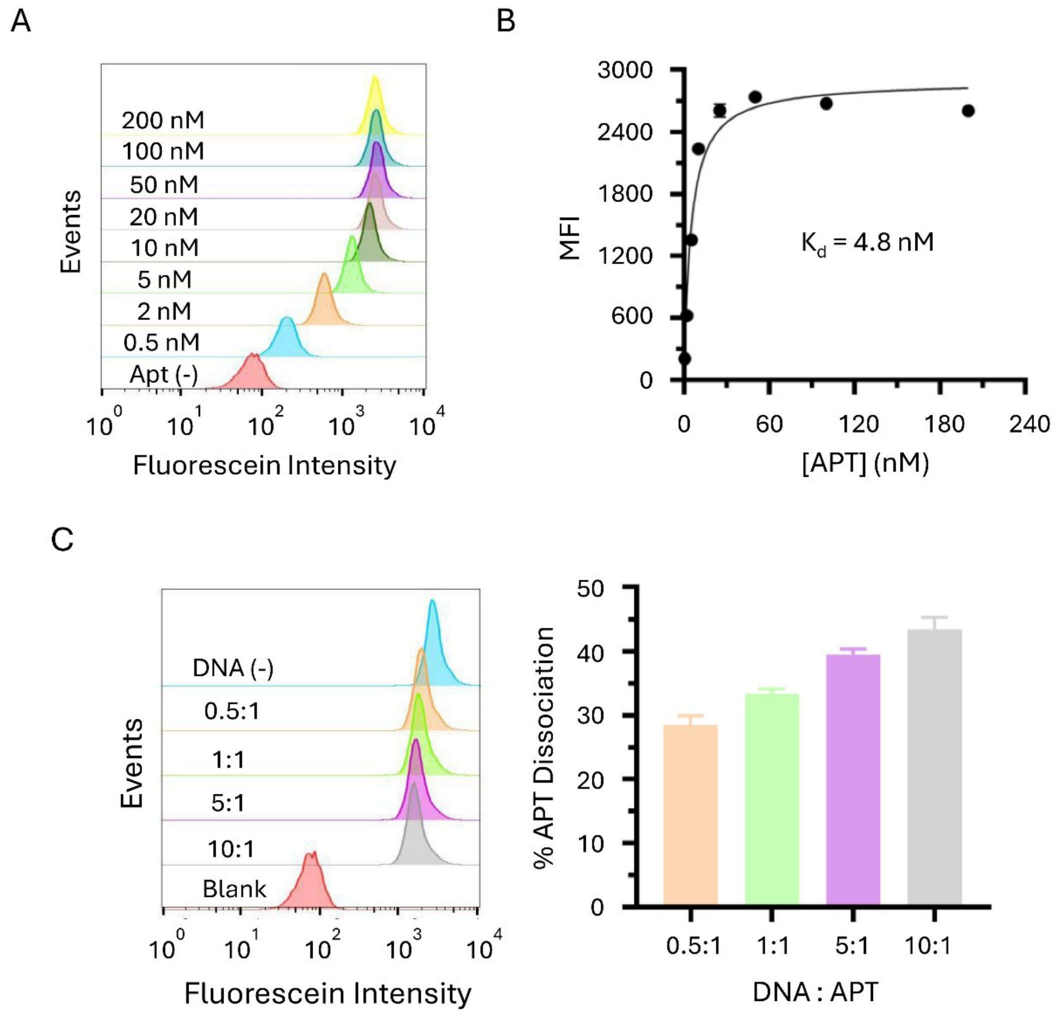

Fig. S1 The binding of hybrid bispecific aptamers without toehold sequence. (A) Flow cytometry of thrombin-bound microparticles treated with varied aptamer concentrations from 0.5 to 200 nM. (B) Fitted curve of aptamer−thrombin binding. MFI, mean fluorescence intensity. APT, aptamer. (C) Flow cytometry analysis of DNA-triggered aptamer dissociation from thrombin on the microparticle surface. The concentration of HBA was 500 nM, and the molar ratio of DNA trigger to aptamer was varied from 0.5:1 to 10:1.

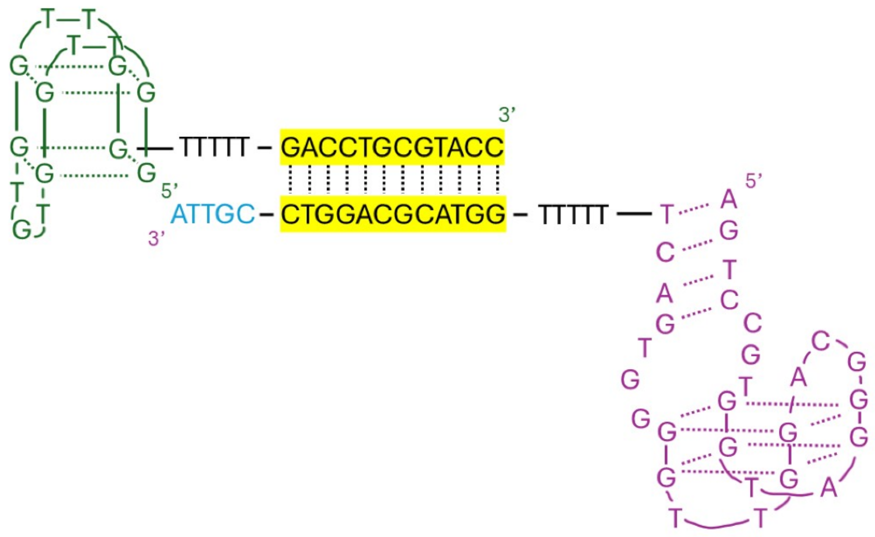

Fig. S2 The structure of hybrid bispecific aptamer with the toehold sequence. The hybrid bispecific aptamer is a hybridized product of an exosite I-targeting (anti-exosite I) strand and an exosite II–targeting (anti-exosite II) strand. The anti-exosite I strand contains 15-mer (in green) and a sequence (highlighted) for hybridization with anti-exosite II strand, which were separated by 5T spacer. The anti-exosite II strand contains a hybridization sequence (highlighted) and 29-mer (in purple), separated by 5T spacer. The sequence in blue is the toehold sequence on the anti-exosite II strand.

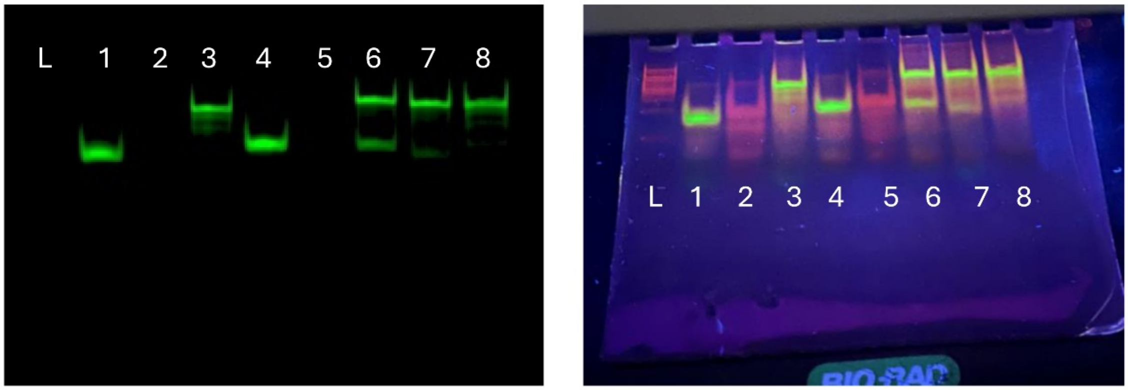

Fig. S3 Gel electrophoresis for evaluation of hybridization efficiency of bivalent hybrid aptamers with vs. without toehold sequence. Left: PAGE image showing fluorescence from FAM-labeled exosite I-targeting strands (15-mer-5T-Sticky & 15-mer-5T-Sticky-Toehold). Right: PAGE image showing the ladder and exosite II-targeting strand stained with SYBR Safe (red). 1, 15-mer-5T-Sticky. 2, 29-mer-5T-Sticky. 3, Hybridized product of 1 and 2. 4, 15-mer-5T-Sticky-Toehold. 5, 29-mer-5T-Sticky-Toehold. 6, hybridized product of 4 and 5. 7, hybridized product of 1 and 5. 8, hybridized product of 2 and 4. L, Ladder.

A

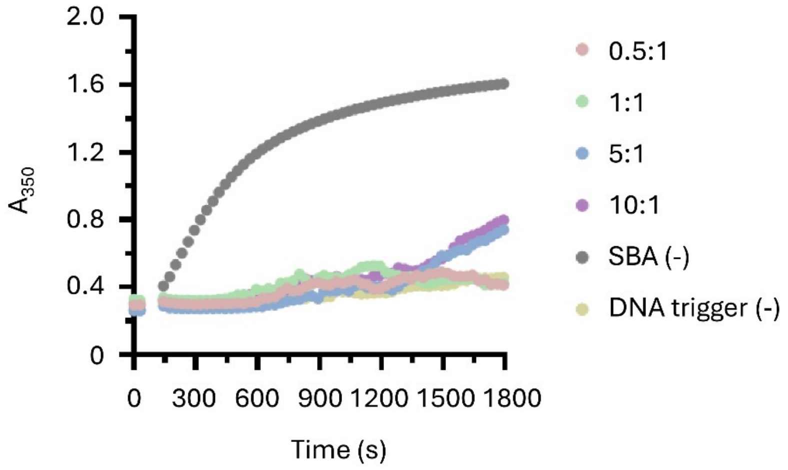

B

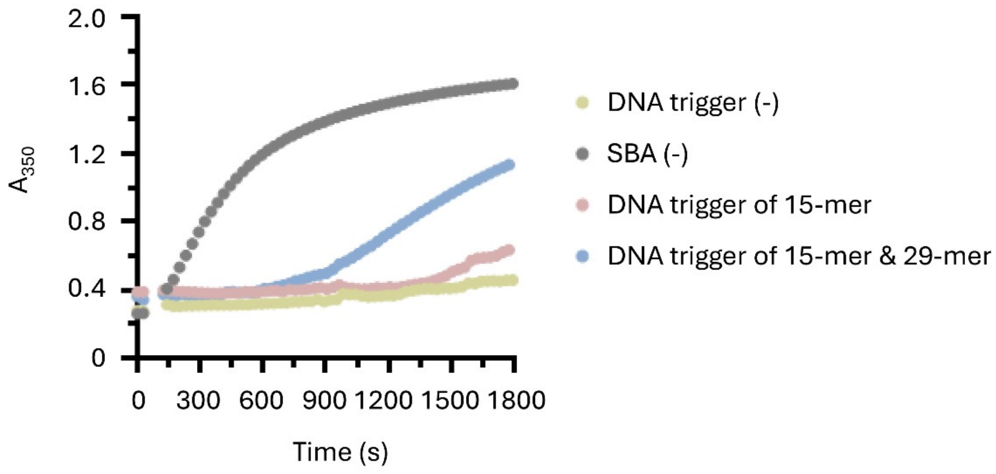

Fig. S4 Effect of DNA trigger on the gelation of fibrin hydrogel inhibited by SBA. (A) Turbidity kinetics of fibrin formation in the presence of SBA (250 nM) and DNA trigger hybridizing the same region as THBA on SBA, at different molar ratio to SBA. Sequence of DNA trigger from 5' to 3': GGACTAAAAAAAAAAAAAAAA. (B) Turbidity kinetics of fibrin formation in the presence of SBA (250 nM) and DNA trigger of 15-mer and 29-mer. The DNA trigger-to-SBA molar ratio was 10:1.

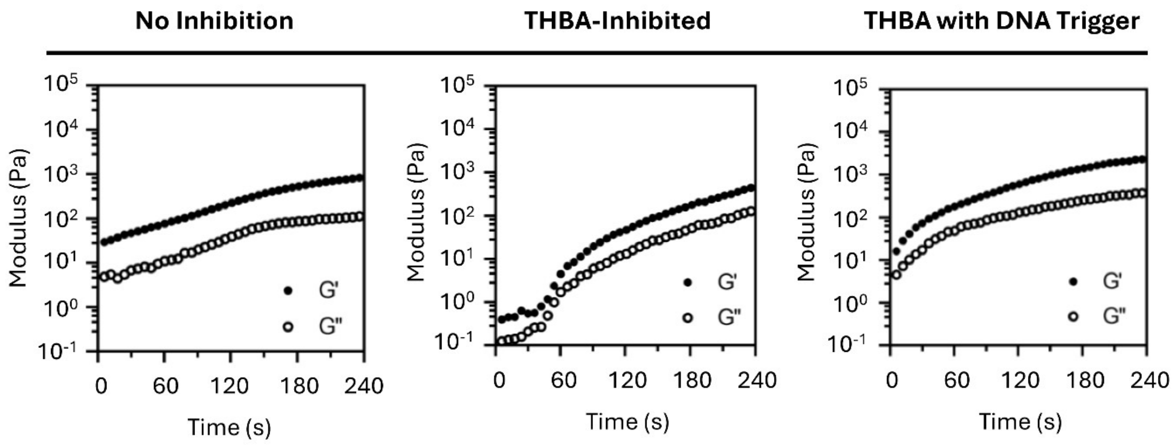

Fig. S5 Rheology of THBA-Inhibited Fibrin Hydrogel with vs. without DNA trigger. A time sweeping test was used to determine the storage (G') and loss (G") moduli. The fibrinogen concentration was 25 mg mL^{-1}, and the thrombin concentration was 2.5 U mL^{-1}. The aptamer concentration was 250 nM. The DNA trigger-to-THBA molar ratio was 10:1.

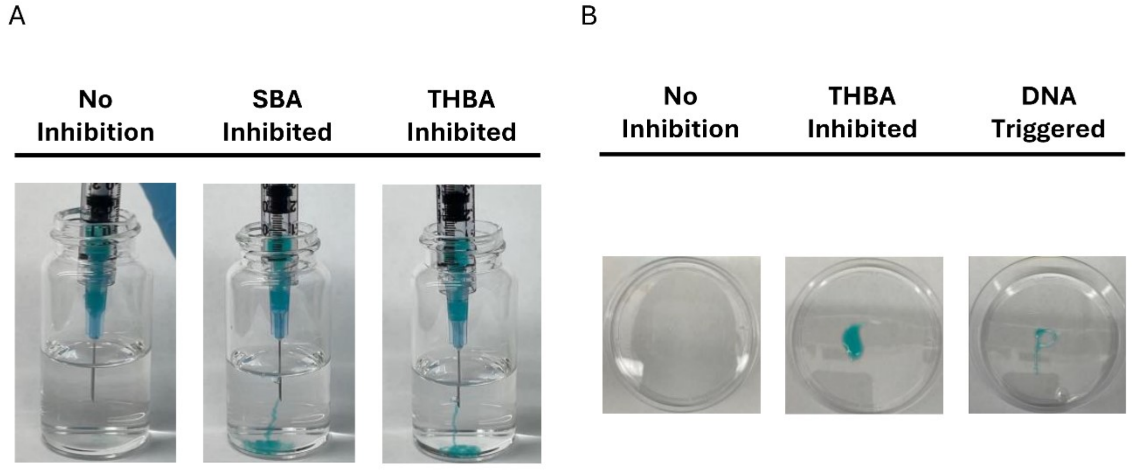

Fig. S6 Assessment on injectability of THBA-inhibited fibrin hydrogel and gelation of inhibited hydrogel with DNA trigger. (A) The injection of pre-gel fibrin solution from the syringe to a vial at 240 s post the solution mixing. (B) The extrusion of pre-gel fibrin solutions from the syringe to a plate surface at 120 s post the solution mixing. The aptamer concentration was 250 nM. The DNA trigger-to-THBA molar ratio was 10:1.
